# Curvilinear regression analysis of benzenoid hydrocarbons and computation of some reduced reverse degree based topological indices for hyaluronic acid-paclitaxel conjugates

**DOI:** 10.1038/s41598-023-28416-3

**Published:** 2023-02-24

**Authors:** Vignesh Ravi, Kalyani Desikan

**Affiliations:** Division of Mathematics, School of Advanced Sciences, Vellore Institute of Technology, Chennai, India

**Keywords:** Applied mathematics, Cheminformatics

## Abstract

Graph theoretical molecular descriptors alias topological indices are a convenient means for expressing in numerical form the chemical structure encoded in a molecular graph. The structure descriptors derived from molecular graphs are widely used in quantitative structure-property relationship (QSPR) and quantitative structure-activity relationship (QSAR) studies. The reason for introducing new indices is to obtain predictions of target properties of considered molecules that are better than the predictions obtained using already known indices. In this paper, we apply the reduced reverse degree based indices introduced in 2021 by Vignesh et al. In the QSPR analysis, we first compute the reduced reverse degree based indices for a family of benzenoid hydrocarbon molecules and then we obtain the correlation with the Physico-chemical properties of the considered molecules. We show that all the properties taken into consideration for the benzenoid hydrocarbons can be very effectively predicted by the reduced reverse degree based indices. Also, we have compared the predictive capability of reduced reverse degree based topological descriptors against 16 existing degree based indices. Further, we compute the defined reduced reverse degree based topological indices for Hyaluronic Acid-Paclitaxel Conjugates $(HAP)_{n}$, $n \ge 1$.

## Introduction

The most important part of the drug discovery process is finding and improving promising lead compounds as quickly and cheaply as possible. Drug design methodologies can be classified into two primary groups: ligand based and ligand-receptor interaction based approaches. Ligand based apporaches depend only on the structure of ligands. Ligand-receptor interaction based approaches are often referred to as structure-based methods (docking, molecular dynamics, etc.).

The past decade has seen tremendous progress in computational approaches to drug design, and this has played a crucial part in the creation of several currently available medications. Molecular topology^1^ is a new approach used to design and select new molecules, particularly new pharmaceuticals. It uses topological factors rather than physical or geometric dimensions. Molecular topology not only provides an alternate method but also a different paradigm compared to conventional drug design methodologies. Molecular topology has proven to be useful in molecular design despite its inability to account for the physical or chemical properties of ligands and receptors or the ligand-receptor interaction. In drug development and design, molecular topology has been used to find new hits and leads. The discovery of novel lead compounds using molecular topology has enabled the interpretation of results in terms of their structural and physicochemical properties. The toxicity profile of a drug can be affected by its physicochemical properties, which are thought to play a role in its absorption, distribution, metabolism, and elimination. Physicochemical descriptors form the basis of most current approaches to molecular and drug design^2,3^. In this manner, a direct connection can be established between the experimental properties and chemical structures.

A common critique of molecular topology, however, is that it functions like a “black box,” bypassing the mechanism of action that offers neither a physical explanation nor a chemical explanation of the processes^4^. However, this critique may be answered by the fact that topology determines physical and geometrical magnitudes, a claim backed by both experimental and theoretical physicists. This results in molecular topology becoming a self-consistent and independent paradigm describing the molecular behavior. The results of molecular topology can be understood both in terms of structure and in terms of physicochemical characteristics This leads to both the mechanisms of action and the underlying physical chemistry to be deduced from the mathematical topological pattern. This is the most rational explanation, as it is the physical variable that is dependent on mathematics, not the other way around.

Molecular topology is independent of physicochemical molecular descriptors. This fact explains why it is feasible to find new lead compounds using only the knowledge gleaned from mathematical-topological patterns and to interpret the findings in terms of their structural and physicochemical properties. Molecular Topology has transformed from a potential intriguing option to a cornerstone of the drug discovery process. One of them is Quantitative Structure Analysis Relationship (QSAR), which is still effective and common and is also coupled with machine learning^5–8^. Additionally, molecular topology has become more relevant recently in terms of novel or unexpected uses. For instance, Speck-Planche et al. developed the multi-scale de novo drug design paradigm, which provides instructions to create novel compounds with desirable drug-like properties^9^.

In general, descriptors are classified into two types, namely, Experimental (logP, aqueous solubility etc.,) and Theoretical (assessed in Silico from 1D, 2D or 3D molecular structures) descriptors^1^. Based on the parameterization type, the descriptors are sorted into distinct categories: geometric, quantum-chemical, electrostatic, constitutional, and topological^10^. Descriptors based on the molecular graph representation are widely used because they incorporate precious chemical information like size, degree of branching, neighbourhood of atoms, flexibility and overall shape. Molecular topological indices can be broken down into structure-explicit descriptors like quantum chemical ones and structure-implicit ones like hydrophobicity or electronic constants ones^11^. Incorporating ideas from molecular topology makes computing these indices straightforward. In fact, many physicochemical and biological properties can be quickly and accurately estimated using molecular topology^12^.

Based on the known features of existing pharmaceuticals, topological indices can be used to design new lead compounds. These medications have superior pharmacokinetic, pharmacodynamic and toxicological qualities compared to those already available. This can be accomplished at a very low computational cost and economic cost within a short period of time. Due to the need to reduce the costs associated with both the synthesis and clinical testing of pharmaceuticals, the pharmaceutical industry has contributed to the increased interest in molecular descriptors. The development of the predictive quantitative structure-property relationship (QSPR) and quantitative structure-activity relationship^13^ (QSAR) models is particularly important for the design of fine chemicals and pharmaceuticals that are made for a specific purpose. For Structure Activity Relationship (SAR) connections to be developed for medicinal compounds using computational or theoretical methods, accurate representations of molecular structure are essential. The molecular descriptor is the final outcome of applying a logical and mathematical procedure that enables the transformation of chemical information encoded in the symbolic representation of a molecule into a meaningful number.

Lead discovery and lead optimization are only two examples of how topological indices are put to use in the drug design and discovery process. Topological indices play an important part in the process of developing any QSPR or QSAR^14^ model because they quantitatively represent the chemical information that is encoded. They allow researchers to delve into the mechanical aspects of a biochemical process and aid in uncovering the mathematical correlation that exists between chemical structure information and the response of interest. For instance, natural bio-polymer monomers (nucleotides and amino acids) can act as nodes in a graph, with the edges representing covalent bonds, hydrogen bridges, electrostatic interactions, and van der Waals bonds. As a result, the structure of complex biopolymers can be reduced to the topology of a graph, revealing useful information about these molecular systems. Topological indices can be used to characterize graphs that represent molecular systems. Topological indices store information about the connections between molecular atoms and the properties of those atoms. Natural biopolymers such as DNA, RNA, and protein sequences can also be characterized in this manner.

Considering that a theoretical physics starting hypothesis was proved for the first time in experimental physics, the following result is pertinent^4^. Traditionally it has been believed that topology is dependent on energy. This false assumption gave rise to the criticism that topological descriptors serve as a “black box” since they lack a physical interpretation. Now, if topology (a mathematical magnitude) is what determines energy (a physical magnitude) rather than the other way around, as advanced physicists and chemists claim, then mathematical descriptors can be used to predict the energy of any system along with other physical or experimental properties dependent on it. This is precisely what happens when novel active molecules are created or found utilising topological indices.

Quantitative structure-property relationship (QSPR) and Quantitative structure-activity relationship (QSAR)^15^ studies rely heavily on molecular descriptors, many of which are based on topological indices.

In a chemical graph, nodes represent atoms or molecules and the links denote the chemical bonding between the atoms or molecules. Graph theoretical molecular descriptors are topological indices. These are graph invariants that play an important role in pharmaceutical science, chemistry, materials science and engineering, etc. The value of a molecular descriptor is not dependent on the particular molecular representation, such as atom numbering or labeling. Molecules of hydrocarbons are modeled using the corresponding molecular graph. Here the vertices represent the carbon atoms and the edges represent the bonds between them. The degree of a vertex represents the number of edges that are incident on that vertex and it is denoted by $d_{u}$ or *d*(*u*)^16^.

Benzenoid hydrocarbons are represented through benzenoid graphs comprising of hexagons. Here there are only two types of vertices, (*i*.*e*.), vertices of degree 2 or 3^17,18^. In benzenoids, there are only 3 types of edges, (*i*.*e*.), (2, 2), (2, 3) and (3, 3) where the numbers in each tuple denote the degree of the end vertices of the corresponding edge. Twelve physico-chemical properties of benzenoid hydrocarbons have been selected based on the availability of data: Boiling Point (*BP*), Critical Temperature (*CT*), Critical Volume (*CV*), Critical Pressure (*CP*), Exact Mass, Heavy Atom Count, *HL*, *GE*, *Log* *P*, *MR*, *PI* and Molecular Weight (*MW*). The data for the benzenoid hydrocarbon molecules is presented in Table 1. Energy of benzenoid hydrocarbons are taken from^19^. The experimental values of boiling points were taken from Basak et al.^20^.

Vignesh et al.^21^ defined the reduced reverse degree as

1 $$\begin{aligned} \mathcal{R}\mathcal{R}(v) = \Delta (G) - d(v) + 2 \end{aligned}$$

  Vignesh et al.^21^ proposed some reduced reverse degree-based topological indices. These indices are

2 $$\begin{aligned}{} & {} \mathcal{R}\mathcal{R}M_{1}(G) = \sum \limits _{u v \in E} \Big [\mathcal{R}\mathcal{R}(u) + \mathcal{R}\mathcal{R}(v) \Big ] \end{aligned}$$

3 $$\begin{aligned}{} & {} \mathcal{R}\mathcal{R}M_{2}(G) = \sum \limits _{u v \in E} \Big [\mathcal{R}\mathcal{R}(u) * \mathcal{R}\mathcal{R}(v) \Big ] \end{aligned}$$

4 $$\begin{aligned}{} & {} \mathcal{R}\mathcal{R}HM_{1}(G) = \sum \limits _{u v \in E} \Big [\mathcal{R}\mathcal{R}(u) + \mathcal{R}\mathcal{R}(v) \Big ]^{2} \end{aligned}$$

5 $$\begin{aligned}{} & {} \mathcal{R}\mathcal{R}HM_{2}(G) = \sum \limits _{u v \in E} \Big [\mathcal{R}\mathcal{R}(u) * \mathcal{R}\mathcal{R}(v) \Big ]^{2} \end{aligned}$$

6 $$\begin{aligned}{} & {} \mathcal{R}\mathcal{R}F(G) = \sum \limits _{u v \in E} \Big [\mathcal{R}\mathcal{R}(u)^{2} + \mathcal{R}\mathcal{R}(v)^{2} \Big ] \end{aligned}$$

7 $$\begin{aligned}{} & {} \mathcal{R}\mathcal{R}ABC(G) = \sum \limits _{u v \in E} \Bigg [\sqrt{\frac{\mathcal{R}\mathcal{R}(u) + \mathcal{R}\mathcal{R}(v) - 2}{\mathcal{R}\mathcal{R}(u) * \mathcal{R}\mathcal{R}(v)}} \Bigg ] \end{aligned}$$

8 $$\begin{aligned}{} & {} \mathcal{R}\mathcal{R}GA(G) = \sum \limits _{u v \in E} \Bigg [\frac{2\sqrt{\mathcal{R}\mathcal{R}(u) * \mathcal{R}\mathcal{R}(v)}}{\mathcal{R}\mathcal{R}(u) + \mathcal{R}\mathcal{R}(v)} \Bigg ] \end{aligned}$$

9 $$\begin{aligned}{} & {} \mathcal{R}\mathcal{R}R_{\alpha }(G) = \sum \limits _{u v \in E} \Big [\mathcal{R}\mathcal{R}(u) * \mathcal{R}\mathcal{R}(v) \Big ]^{\alpha } \end{aligned}$$

In Eq. (9) we consider $\alpha = 1, -1, \frac{1}{2}, \frac{-1}{2}$.

In this proposed work, we consider 26 benzenoid hydrocarbon molecules and obtain the values for the above-mentioned reduced reverse degree based topological indices to perform curvilinear analysis and ascertain the predictive capability of the indices against the physico-chemical properties of the benzenoid hydrocarbons.

## Curvilinear regression analysis of proposed indices

In this section, we analyze the aforesaid reduced reverse degree based topological indices with respect to the following physico-chemical properties of the benzenoid hydrocarbon molecules: Boiling Point (*BP*), Critical Pressure (*CP*), Critical Temperature (*CT*), Critical Volume (*CV*), Exact Mass, Heavy Atom Count, *HL*, *GE*, *Log* *P*, *MR*, *PI* and Molecular Weight (*MW*). The experimental values of physico-chemical properties of benzenoid hydrocarbon molecules are given in Table 1.

**Table 1:** Data for benzenoid hydrocarbons.

S. No.	BP	CT	CP	CV	GE	LOG P	MR	HL	PI	MW	EM	HAC
1	80.1	323.79	47.69	263.5	121.68	2.03	25.28	0.66	8	78.11	78.04695	6
2	218	484.95	38.97	409.5	252.38	3.03	42.45	1.67	13.6832	128.17	128.0626	10
3	340	586.11	32.43	555.5	383.08	4.03	59.62	2.68	19.3137	178.23	178.0783	14
4	340	586.11	32.43	555.5	383.08	4.03	59.62	2.68	19.4483	178.23	178.0783	14
5	432.02	650.8	27.41	701.5	513.78	5.9	76.79	3.69	24.9308	228.3	228.0909	18
6	436.7	650.8	27.41	701.5	513.78	5.03	76.79	3.69	25.1875	228.3	228.0909	18
7	438	650.8	27.41	701.5	513.78	5.03	76.79	3.69	25.1012	228.3	228.0909	18
8	448	650.8	27.41	701.5	513.78	5.03	76.79	3.69	25.1922	228.3	228.0909	18
9	438	650.8	27.41	701.5	513.78	5.03	76.79	3.69	25.2745	228.3	228.0909	18
10	404	625.65	30.73	619.5	491.18	5.08	68.36	3.47	22.5055	202.25	202.0783	16
11	524.6	714.53	23.47	847.5	644.48	6.02	93.96	4.7	30.544	278.3	278.1096	22
12	547.5	714.53	23.47	847.5	644.48	6.02	93.96	4.7	30.7255	278.3	278.1096	22
13	524	714.53	23.47	847.5	644.48	6.02	93.96	4.7	30.8805	278.3	278.1096	22
14	524.7	714.53	23.47	847.5	644.48	6.02	93.96	4.7	30.8795	278.3	278.1096	22
15	547.5	714.53	23.47	847.5	644.48	6.02	93.96	4.7	30.7627	278.3	278.1096	22
16	525	714.53	23.47	847.5	644.48	6.02	93.96	4.7	30.999	278.3	278.1096	22
17	524.7	714.53	23.47	847.5	644.48	6.02	93.96	4.7	30.9386	278.3	278.1096	22
18	520	714.53	23.47	847.5	644.48	6.02	93.96	4.7	30.9432	278.3	278.1096	22
19	524.7	714.53	23.47	847.5	644.48	6.02	93.96	4.7	30.839	278.3	278.1096	22
20	518	714.53	23.47	847.5	644.48	6.02	93.96	4.7	30.9418	278.3	278.1096	22
21	400	689.17	26.08	765.5	621.88	5.34	85.53	4.48	28.2453	252.3	252.0939	20
22	467.5	689.17	26.08	765.5	621.88	5.34	85.53	4.48	28.3361	252.3	252.0939	20
23	495	689.17	26.08	765.5	621.88	5.34	85.53	4.48	28.222	252.3	252.0939	20
24	500	728.06	24.85	829.5	729.98	5.66	94.28	5.27	31.4251	276.3	276.0939	22
25	604	779.67	20.33	993.5	775.18	7.02	111.13	5.71	36.1557	328.4	328.1252	26
26	525	767.68	23.7	893.5	838.08	5.98	103.02	6.06	34.5718	300.4	300.0939	24

We analyze the topological indices via-a-vis the physico-chemical properties using the following regression models

10 $$\begin{aligned}{} & {} P = \alpha _{1}(TI)+\gamma \end{aligned}$$

11 $$\begin{aligned}{} & {} P = \alpha _{1}(TI)^{2}+\alpha _{2}(TI)+\gamma \end{aligned}$$

where *P* is the physical property, TI is the topological descriptor, $\alpha _{i}$, $i = 1, 2$ and $\gamma$ represent the coefficients and constant, respectively. For the twelve physico-chemical properties we found the correlation between the properties and the twenty indices proposed by us. Based on the recommendations of the International Academy of Mathematical Chemistry (IAMC), we have only considered the topological indices for which $R^{2} \ge 0.8$. We now present the analysis of the linear and quadratic regression models based on the $R^{2}$ value and Root Mean Square Error value.

## Results and discussion

Using Eq. (10), we obtained the linear regression models for the physico-chemical properties vis-a-vis the index for which the $R^{2}$ value is maximum for the property. We observe that

- $\mathcal{R}\mathcal{R}R_{-1}$ is best suited for predicting Boiling Point (*BP*), Critical Pressure (*CP*) and *Log* *P* with corresponding $R^{2}$ values 0.9587, 0.9539 and 0.9637, respectively.
- $\mathcal{R}\mathcal{R}R_{\frac{-1}{2}}$ is best suited for predicting Critical Volume (*CV*), Exact Mass (*EM*), Heavy Atom Count (*HAC*), Molar Refraction (*MR*) and Molecular Weight (*MW*) with corresponding $R^{2}$ values 0.9967, 0.9979, 0.9979, 0.9979 and 0.9979, respectively.
- $\mathcal{R}\mathcal{R}HM_{1}$ is best suited for predicting (*GE*) with $R^{2}$ value 0.9986.
- $\mathcal{R}\mathcal{R}GA$ is best suited for predicting Critical Temperature (*CT*) with $R^{2}$ value 0.9602.
- $\mathcal{R}\mathcal{R}ABC$ is best suited for predicting (*PI*) with $R^{2}$ value 0.9976.
- $\mathcal{R}\mathcal{R}F$ is best suited for predicting Henry’s Law (*HL*) with $R^{2}$ value 0.9995.

Hence the linear regression models for predicting Boiling Point (*BP*), Critical Pressure (*CP*), Critical Temperature (*CT*), Critical Volume (*CV*), Exact Mass, Heavy Atom Count, *HL*, *GE*, *Log* *P*, *MR*, *PI* and Molecular Weight (*MW*) are:

12 $$\begin{aligned}{} & {} \widehat{BP} = 129(\mathcal{R}\mathcal{R}R_{-1})-75.26 \end{aligned}$$

13 $$\begin{aligned}{} & {} \widehat{Log P} = 1.228(\mathcal{R}\mathcal{R}R_{-1})+0.296 \end{aligned}$$

14 $$\begin{aligned}{} & {} \widehat{CT} = 16.62(\mathcal{R}\mathcal{R}GA)+291.4 \end{aligned}$$

15 $$\begin{aligned}{} & {} \widehat{GE} = 0.9654(\mathcal{R}\mathcal{R}HM_{1})+26.46 \end{aligned}$$

16 $$\begin{aligned}{} & {} \widehat{PI} = 1.63(\mathcal{R}\mathcal{R}ABC)+1.197 \end{aligned}$$

17 $$\begin{aligned}{} & {} \widehat{HL} = 0.01448(\mathcal{R}\mathcal{R}F)-0.03858 \end{aligned}$$

18 $$\begin{aligned}{} & {} \widehat{CP} = -6.6085(\mathcal{R}\mathcal{R}R_{-1})+54.162 \end{aligned}$$

19 $$\begin{aligned}{} & {} \widehat{CV} = 73.21\Big (\mathcal{R}\mathcal{R}R_{\frac{-1}{2}}\Big )+46.92 \end{aligned}$$

20 $$\begin{aligned}{} & {} \widehat{EM} = 25.23\Big (\mathcal{R}\mathcal{R}R_{\frac{-1}{2}}\Big )+3.109 \end{aligned}$$

21 $$\begin{aligned}{} & {} \widehat{HAC} = 2.024\Big (\mathcal{R}\mathcal{R}R_{\frac{-1}{2}}\Big )-0.022 \end{aligned}$$

22 $$\begin{aligned}{} & {} \widehat{MR} = 8.699\Big (\mathcal{R}\mathcal{R}R_{\frac{-1}{2}}\Big )-0.6273 \end{aligned}$$

23 $$\begin{aligned}{} & {} \widehat{MW} = 25.25\Big (\mathcal{R}\mathcal{R}R_{\frac{-1}{2}}\Big )+3.143 \end{aligned}$$

  We use Eq. (12) to compute the predicted boiling point $\widehat{BP}$ for each of the benzenoid hydrocarbon molecules, refer Table 2. Among the linear models, we have listed the best predictive models for the remaining physical properties in Eqs. (13) to (23).

We obtain the predicted values for all properties using the proposed linear models and we summarize the results in Table 3. From Table 3 we see that the average residual error of *BP* is $5.3008 \%$ with $R^{2}$ value 0.9587.

**Table 2:** Predicted boiling points for 26 benzenoid hydrocarbon molecules.

S. No.	$\mathcal{R}\mathcal{R}R_{-1}$	BP	$\widehat{BP}$	RES	$RES^{2}$	$RES^{2} / BP$	RES %
1	1.5	80.1	118.24	38.14	1454.66	18.16054	47.61548
2	2.2778	218	218.5762	0.5762	0.332006	0.001523	0.264312
3	3.0556	340	318.9124	21.0876	444.6869	1.307903	6.202235
4	3.0833	340	322.4857	17.5143	306.7507	0.902208	5.151265
5	3.8333	432.02	419.2357	12.7843	163.4383	0.378312	2.959192
6	3.8889	436.7	426.4081	10.2919	105.9232	0.242554	2.356744
7	3.8611	438	422.8219	15.1781	230.3747	0.52597	3.46532
8	3.8889	448	426.4081	21.5919	466.2101	1.040648	4.819621
9	3.9167	438	429.9943	8.0057	64.09123	0.146327	1.827785
10	3.8889	404	426.4081	22.4081	502.1229	1.242879	5.546559
11	4.6111	524.6	519.5719	5.0281	25.28179	0.048193	0.958464
12	4.6389	547.5	523.1581	24.3419	592.5281	1.082243	4.446009
13	4.6667	524	526.7443	2.7443	7.531182	0.014372	0.523721
14	4.6667	524.7	526.7443	2.0443	4.179162	0.007965	0.389613
15	4.6389	547.5	523.1581	24.3419	592.5281	1.082243	4.446009
16	4.7222	525	533.9038	8.9038	79.27765	0.151005	1.695962
17	4.6944	524.7	530.3176	5.6176	31.55743	0.060144	1.070631
18	4.6944	520	530.3176	10.3176	106.4529	0.204717	1.984154
19	4.6667	524.7	526.7443	2.0443	4.179162	0.007965	0.389613
20	4.6944	518	530.3176	12.3176	151.7233	0.292902	2.377915
21	4.2222	400	469.4038	69.4038	4816.887	12.04222	17.35095
22	3.9444	467.5	433.5676	33.9324	1151.408	2.462904	7.258267
23	4.1944	495	465.8176	29.1824	851.6125	1.720429	5.895434
24	4.5278	500	508.8262	8.8262	77.90181	0.155804	1.76524
25	5.3889	604	619.9081	15.9081	253.0676	0.418986	2.633791
26	4.8333	525	548.2357	23.2357	539.8978	1.028377	4.425848

The hypotheses which we considered for checking the goodness of fit of the regression models are:

$$H_{0}:$$
Proposed regression model is a good fit.
$$H_{1}:$$
Proposed regression model is not a good fit.

The table value for goodness of fit ($\chi ^{2}$) with d.o.f. 25 is 37.652 for $5 \%$ level of significance.

**Table 3:** Statistical summary of linear models for 26 benzenoid hydrocarbons.

Property	Model number	$R^{2}$	$\chi ^{2}$	Avg. residual $\%$	RMSE
*BP*	(12)	0.9587	44.7293	5.3008	23.2957
*Log* *P*	(13)	0.9637	0.18095	1.9591	0.2074
*CT*	(14)	0.9608	20.3819	2.3567	18.9922
*GE*	(15)	0.9986	1.3062	0.7285	5.8237
*PI*	(16)	0.9976	0.0855	0.8416	0.3184
*HL*	(17)	0.9995	0.0035	0.2915	0.0256
*CP*	(18)	0.9539	1.2389	3.1242	1.2647
*CV*	(19)	0.9967	2.6089	0.7031	9.3284
*EM*	(20)	0.9979	0.6239	0.4748	2.5671
*HAC*	(21)	0.9979	0.0505	0.4404	0.2046
*MR*	(22)	0.9978	0.2287	0.4379	0.9010
*MW*	(23)	0.9979	5.0938	0.4796	2.5666

From Table 3, we observe that the calculated $\chi ^{2}$ values for all the properties except *BP* are lesser than the table value with d.o.f. 25 for $5 \%$ level of significance. Thus we accept all the models except model (12).

Though the $R^{2}$ values are high in the linear models, some of the models have high residual error and thus we explore the predictive capability of quadratic regression models.

## Quadratic regression models

Using Eq. (11), we obtained the quadratic regression models for the physico-chemical properties vis-a-vis each of the proposed indices. We observe that

- $\mathcal{R}\mathcal{R}R_{-1}$ is best suited for predicting Boiling Point (*BP*) and *Log* *P* with corresponding $R^{2}$ values 0.9673 and 0.9651, respectively.
- $\mathcal{R}\mathcal{R}R_{\frac{-1}{2}}$ is best suited for predicting Critical Pressure (*CP*), Critical Volume (*CV*), Exact Mass (*EM*), Heavy Atom Count (*HAC*), Molar Refraction (*MR*) and Molecular Weight (*MW*) with corresponding $R^{2}$ values 0.9916, 0.9968, 0.9980, 0.9979, 0.9980 and 0.9980, respectively.
- $\mathcal{R}\mathcal{R}HM_{1}$ is best suited for predicting (*GE*) with $R^{2}$ value 0.9988.
- $\mathcal{R}\mathcal{R}GA$ is best suited for predicting Critical Temperature (*CT*) with $R^{2}$ value 0.9918.
- $\mathcal{R}\mathcal{R}ABC$ is best suited for predicting (*PI*) with $R^{2}$ value 0.9978.
- $\mathcal{R}\mathcal{R}F$ is best suited for predicting Henry’s Law (*HL*) with $R^{2}$ value 0.9996.

Among the quadratic models, we listed the best predictive models from Eqs. (24) to (35) and the quadratic regression models for Boiling Point (*BP*), Critical Pressure (*CP*), Critical Temperature (*CT*), Critical Volume (*CV*), Exact Mass, Heavy Atom Count, *HL*, *GE*, *Log* *P*, *MR*, *PI* and Molecular Weight (*MW*) are:

24 $$\begin{aligned}{} & {} \widehat{BP} = -10.47(\mathcal{R}\mathcal{R}R_{-1})^{2} + 202.4(\mathcal{R}\mathcal{R}R_{-1})-192.6 \end{aligned}$$

25 $$\begin{aligned}{} & {} \widehat{Log P} = -0.0407(\mathcal{R}\mathcal{R}R_{-1})^{2} + 1.513(\mathcal{R}\mathcal{R}R_{-1})-0.1598 \end{aligned}$$

26 $$\begin{aligned}{} & {} \widehat{CT} = -0.3939(\mathcal{R}\mathcal{R}GA)^{2} + 31.57(\mathcal{R}\mathcal{R}GA)-166.2 \end{aligned}$$

27 $$\begin{aligned}{} & {} \widehat{GE} = 0.000047(\mathcal{R}\mathcal{R}HM_{1})^{2} + 0.9208(\mathcal{R}\mathcal{R}HM_{1})+35.4 \end{aligned}$$

28 $$\begin{aligned}{} & {} \widehat{PI} = -0.004891(\mathcal{R}\mathcal{R}ABC)^{2} + 1.761(\mathcal{R}\mathcal{R}ABC)+0.4245 \end{aligned}$$

29 $$\begin{aligned}{} & {} \widehat{HL} = 8.815*(10^{-8})(\mathcal{R}\mathcal{R}F)^{2} + 0.01444(\mathcal{R}\mathcal{R}F)-0.03427 \end{aligned}$$

30 $$\begin{aligned}{} & {} \widehat{CP} = 0.1774\Big (\mathcal{R}\mathcal{R}R_{\frac{-1}{2}}\Big )^{2} - 5.46\Big (\mathcal{R}\mathcal{R}R_{\frac{-1}{2}}\Big )+62.12 \end{aligned}$$

31 $$\begin{aligned}{} & {} \widehat{CV} = -0.2004\Big (\mathcal{R}\mathcal{R}R_{\frac{-1}{2}}\Big )^{2} + 76.46\Big (\mathcal{R}\mathcal{R}R_{\frac{-1}{2}}\Big )+35.08 \end{aligned}$$

32 $$\begin{aligned}{} & {} \widehat{EM} = -0.05377\Big (\mathcal{R}\mathcal{R}R_{\frac{-1}{2}}\Big )^{2} + 26.1\Big (\mathcal{R}\mathcal{R}R_{\frac{-1}{2}}\Big )-0.06767 \end{aligned}$$

33 $$\begin{aligned}{} & {} \widehat{HAC} = -0.003732\Big (\mathcal{R}\mathcal{R}R_{\frac{-1}{2}}\Big )^{2} + 2.084\Big (\mathcal{R}\mathcal{R}R_{\frac{-1}{2}}\Big )-0.2424 \end{aligned}$$

34 $$\begin{aligned}{} & {} \widehat{MR} = -0.0148\Big (\mathcal{R}\mathcal{R}R_{\frac{-1}{2}}\Big )^{2} + 8.939\Big (\mathcal{R}\mathcal{R}R_{\frac{-1}{2}}\Big )-1.503 \end{aligned}$$

35 $$\begin{aligned}{} & {} \widehat{MW} = -0.0085\Big (\mathcal{R}\mathcal{R}R_{\frac{-1}{2}}\Big )^{2} + 3.042\Big (\mathcal{R}\mathcal{R}R_{\frac{-1}{2}}\Big )-1.157 \end{aligned}$$

   We use Eq. (24) to compute the predicted boiling point $\widehat{BP}$ for each of the every benzenoid hydrocarbon molecules, refer Table 4. From Table 5 we see that the average error is $3.0962 \%$ with the $R^{2}$ value is 0.9673. Similarly, we predict all properties with the help of the quadratic models which we proposed and we summarize the result in Table 5.

**Table 4:** Predicted boiling points for 26 benzenoid hydrocarbon molecules using quadratic model.

S. No.	$\mathcal{R}\mathcal{R}R_{-1}$	BP	$\widehat{BP}$	RES	$RES^{2}$	$RES^{2} / BP$	RES %
1	1.5	80.1	87.4425	7.3425	53.91231	0.673063	9.166667
2	2.2778	218	214.1045	3.895544	15.17526	0.069611	1.786947
3	3.0556	340	328.0983	11.90172	141.6509	0.41662	3.500505
4	3.0833	340	331.9244	8.075636	65.2159	0.191811	2.375187
5	3.8333	432.02	429.4118	2.608238	6.802904	0.015747	0.603731
6	3.8889	436.7	436.1699	0.530137	0.281046	0.000644	0.121396
7	3.8611	438	432.7989	5.201096	27.0514	0.061761	1.187465
8	3.8889	448	436.1699	11.83014	139.9522	0.312393	2.640656
9	3.9167	438	439.5246	1.524638	2.32452	0.005307	0.348091
10	3.8889	404	436.1699	32.16986	1034.9	2.561634	7.962837
11	4.6111	524.6	518.071	6.529046	42.62845	0.081259	1.244576
12	4.6389	547.5	521.0053	26.49469	701.9684	1.282134	4.839212
13	4.6667	524	523.9235	0.076511	0.005854	1.12E-05	0.014601
14	4.6667	524.7	523.9235	0.776511	0.602969	0.001149	0.147991
15	4.6389	547.5	521.0053	26.49469	701.9684	1.282134	4.839212
16	4.7222	525	529.7009	4.70094	22.09884	0.042093	0.895417
17	4.6944	524.7	526.8151	2.115072	4.473532	0.008526	0.403101
18	4.6944	520	526.8151	6.815072	46.44521	0.089318	1.310591
19	4.6667	524.7	523.9235	0.776511	0.602969	0.001149	0.147991
20	4.6944	518	526.8151	8.815072	77.7055	0.150011	1.701751
21	4.2222	400	475.3249	75.32487	5673.837	14.18459	18.83122
22	3.9444	467.5	442.8512	24.64875	607.5609	1.299596	5.27246
23	4.1944	495	472.1479	22.85206	522.2166	1.054983	4.616578
24	4.5278	500	509.1815	9.181534	84.30057	0.168601	1.836307
25	5.3889	604	594.062	9.937986	98.76357	0.163516	1.645362
26	4.8333	525	541.0725	16.07246	258.324	0.492046	3.061421

The table value for the goodness of fit ($\chi ^{2}$) with d.o.f. 25 is 37.652 for $5 \%$ level of significance. The same hypothesis which we considered in linear models apply for the quadratic models too.

**Table 5:** Statistical summary of quadratic models for 26 benzenoid hydrocarbons.

Property	Model number	$R^{2}$	$\chi ^{2}$	Avg. residual $\%$	RMSE
*BP*	(24)	0.9673	24.6097	3.0962	21.1934
*Log* *P*	(25)	0.9651	0.1703	1.9449	0.2077
*CT*	(26)	0.9874	3.3935	1.0299	10.9277
*GE*	(27)	0.9988	1.2211	0.6948	5.6693
*PI*	(28)	0.9978	0.0814	0.8564	0.3075
*HL*	(29)	0.9996	0.0035	0.2806	0.0261
*CP*	(30)	0.9916	0.2814	1.5133	0.5521
*CV*	(31)	0.9968	2.5356	0.6829	9.4338
*EM*	(32)	0.9980	0.6086	0.4829	2.5974
*HAC*	(33)	0.9980	0.0496	0.4394	0.2075
*MR*	(34)	0.9979	0.2255	0.4630	0.9150
*MW*	(35)	0.9980	0.6076	04819	2.5963

From Table 5, we see that the calculated $\chi ^{2}$ value of boiling point is less than the table value with d.o.f. 25 in $5 \%$ level of significances and thus we accept all the models.

The quadratic models (second order) obtained by us are a good fit for predicting all the properties. Hence we stop with the quadratic regression models.

**Figure 1:**
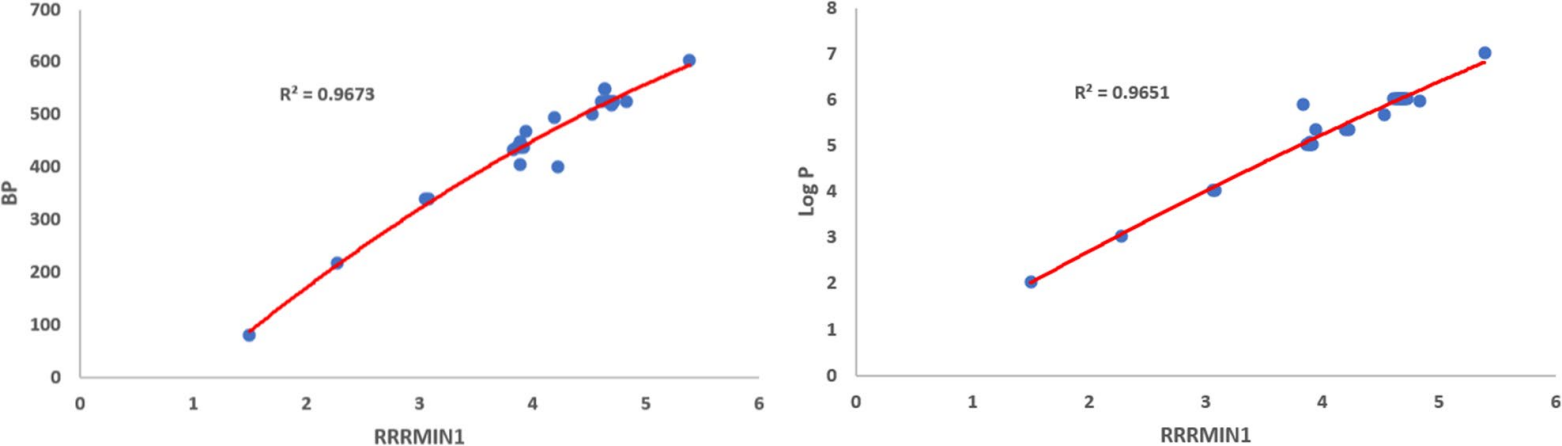
Quadratic regression curves for $\mathcal{R}\mathcal{R}R_{-1}$ against *BP* and *LogP*.

**Figure 2:**
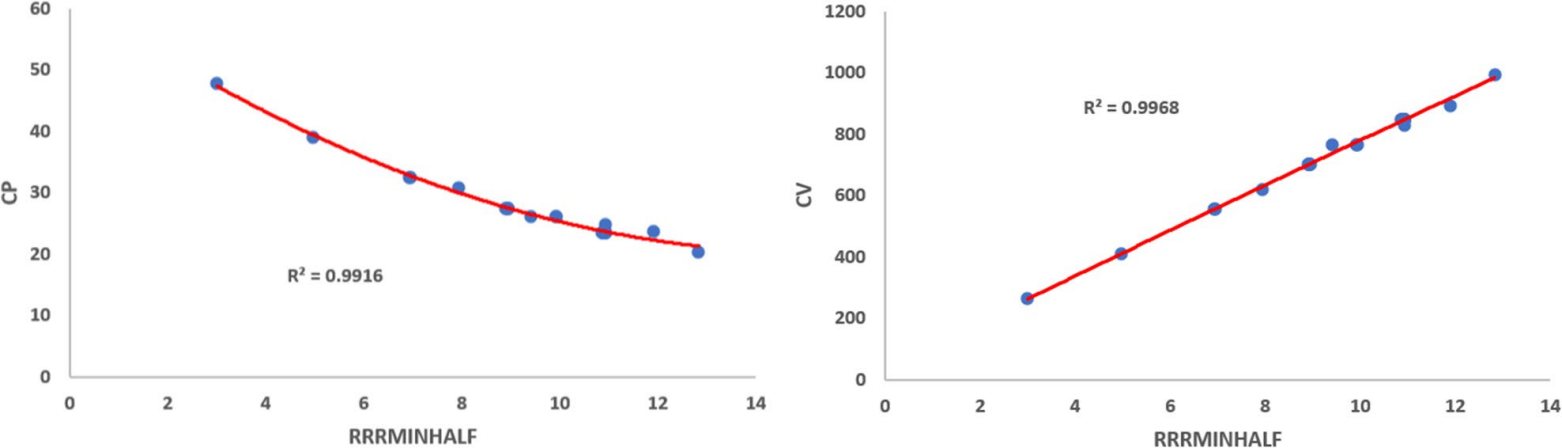
Quadratic regression curves for $\mathcal{R}\mathcal{R}R_{\frac{-1}{2}}$ against *CP* and *CV*.

**Figure 3:**
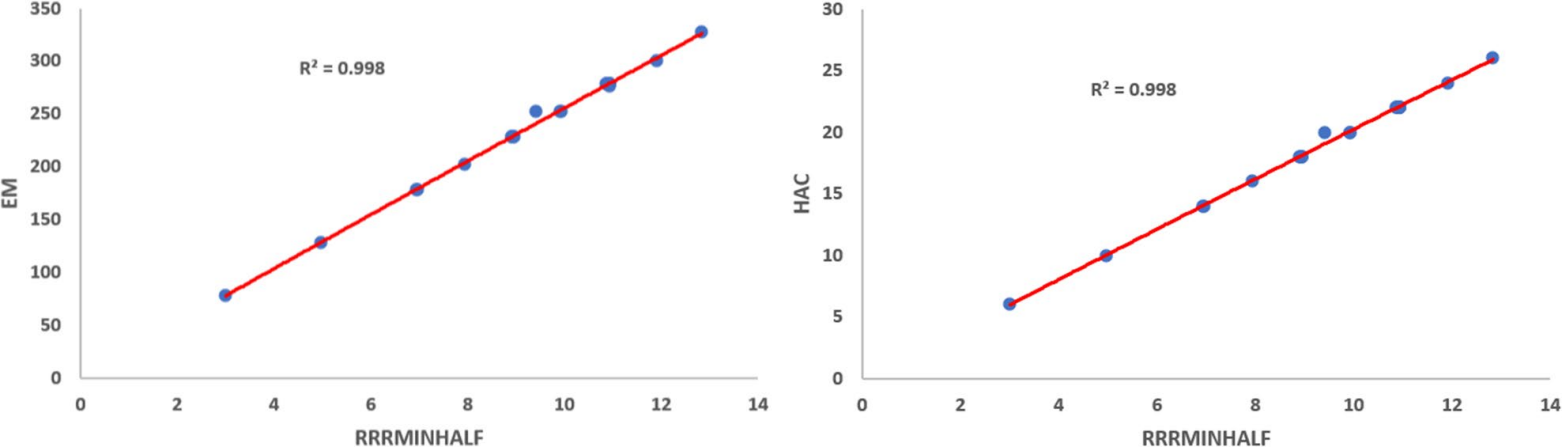
Quadratic regression curves for $\mathcal{R}\mathcal{R}R_{\frac{-1}{2}}$ against *EM* and *HAC*.

**Figure 4:**
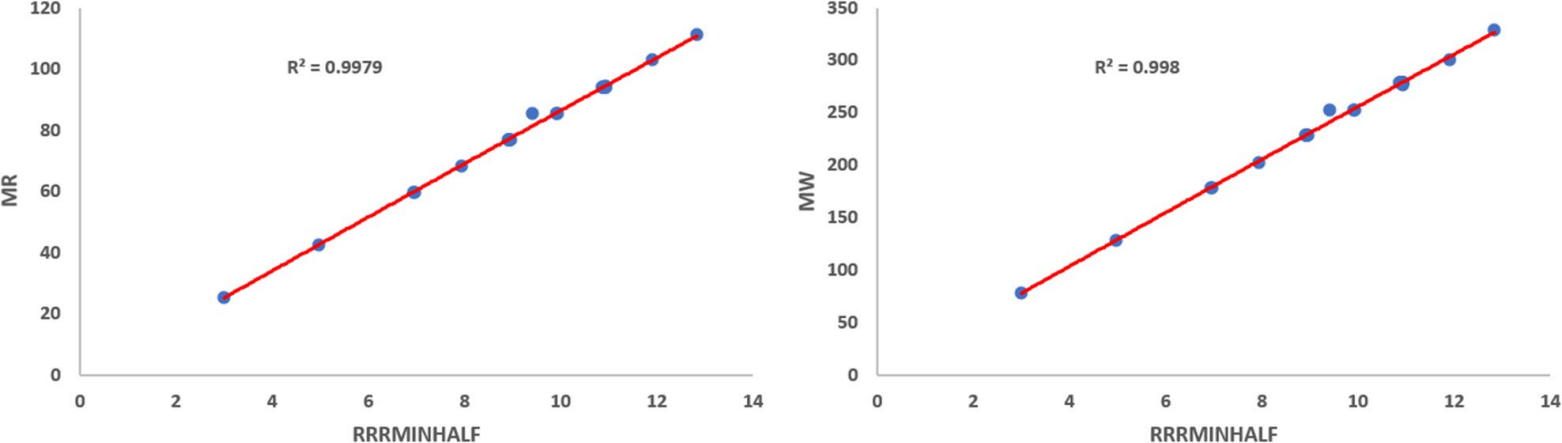
Quadratic regression curves for $\mathcal{R}\mathcal{R}R_{\frac{-1}{2}}$ against *MR* and *MW*.

**Figure 5:**
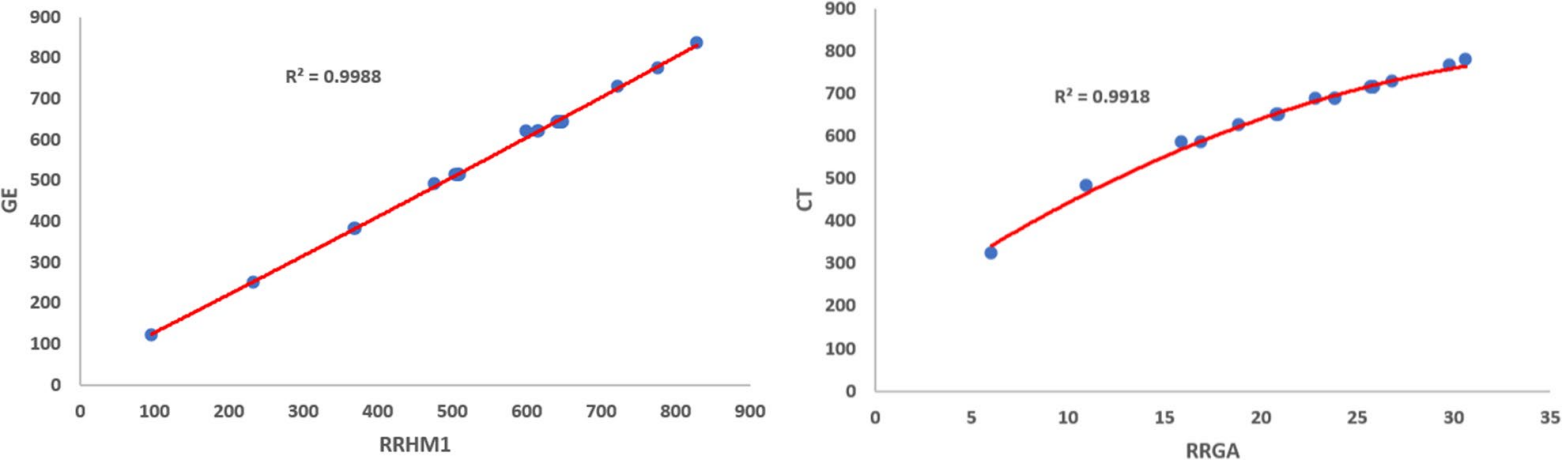
Quadratic regression curves for $\mathcal{R}\mathcal{R}HM_{1}$ against *GE* and $\mathcal{R}\mathcal{R}GA$. against *CT*

**Figure 6:**
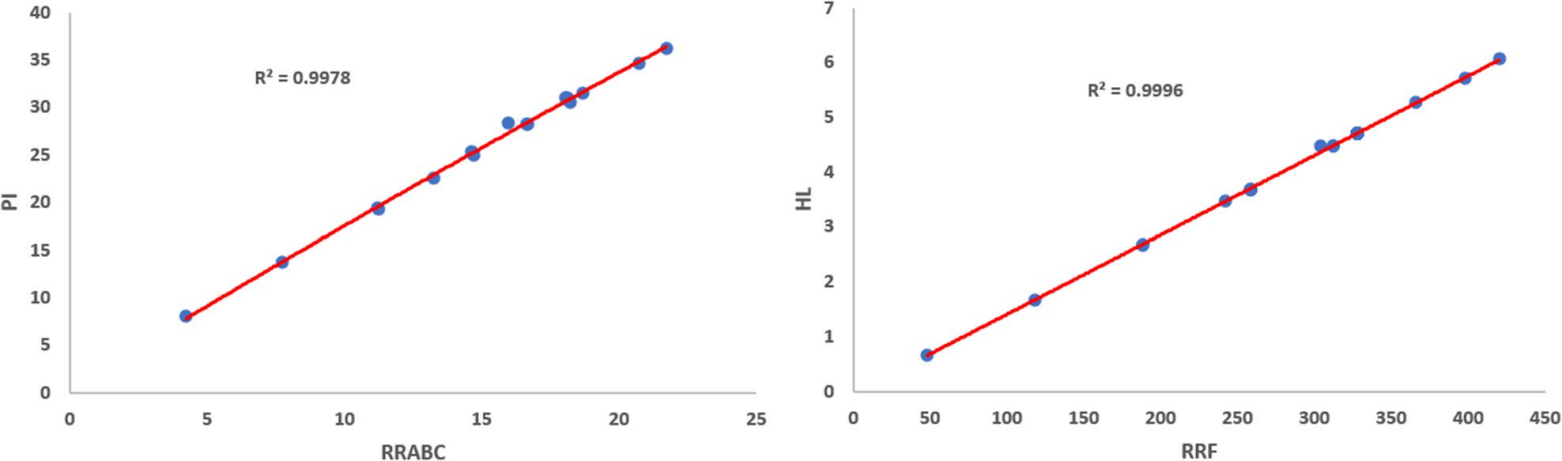
Quadratic regression curves for $\mathcal{R}\mathcal{R}ABC$ against *PI* and $\mathcal{R}\mathcal{R}F$ against *HL*.

Figures 1, 2, 3, 4, 5, 6 show the plots of the quadratic regression models that give the best predicted values for the properties.

## Comparison of our indices against existing indices

In this section, we present the comparison of our proposed indices against 16 well known degree based indices.

The degree based indices that we have taken into consideration include Zagreb ($M_{1}$, $M_{2}$)^22,23^ and Hyper Zagreb ($HM_{1}$, $HM_{2}$)^24,25^ , Forgotten index *F*^26^, Atom Bond Connectivity Index *ABC*^27^, Randić *R*^28^, Geometric Arithmetic index *GA*^29^, Harmonic *H*^30^, Randić version indices $R_{-1}$, $R_{\frac{1}{2}}$ and $R_{\frac{-1}{2}}$, Gourava indices^31^ ($GO_{1}$, $GO_{2}$) and Hyper Gourava indices^32^ ($HGO_{1}$, $HGO_{2}$).

We formulated the linear regression models for the physico-chemical properties vis-a-vis these 16 degree based indices. Among these indices, we selected the best models based on the $R^{2}$ value. For instance, the property Boiling Point (*BP*) is predicted well by the index $R_{\frac{-1}{2}}$. It has a maximum $R^{2}$ value 0.8679.

**Table 6:** Comparison of reduced reverse degree indices against 16 degree based indices.

Property	Existing index	Existing index $R^{2}$	$\mathcal{R}\mathcal{R}$ index/$R^{2}$(linear)	$\mathcal{R}\mathcal{R}$ index /$R^{2}$ (quadratic)
*BP*	$R_{\frac{-1}{2}}$	0.8679	$\mathcal{R}\mathcal{R}R_{-1}$ / 0.9587	$\mathcal{R}\mathcal{R}R_{-1}$ / 0.9673
*Log* *P*	$R_{\frac{-1}{2}}$	0.8629	$\mathcal{R}\mathcal{R}R_{-1}$ / 0.9637	$\mathcal{R}\mathcal{R}R_{-1}$ / 0.9651
*CT*	*ABC*	0.8902	$\mathcal{R}\mathcal{R}GA$ / 0.9608	$\mathcal{R}\mathcal{R}GA$ / 0.9874
*GE*	$GO_{2}$	0.9401	$\mathcal{R}\mathcal{R}HM_{1}$ / 0.9986	$\mathcal{R}\mathcal{R}HM_{1}$ / 0.9988
*PI*	*ABC*	0.9247	$\mathcal{R}\mathcal{R}ABC$ /0.9976	$\mathcal{R}\mathcal{R}ABC$ / 0.9978
*HL*	*F*	0.9392	$\mathcal{R}\mathcal{R}F$ / 0.9995	$\mathcal{R}\mathcal{R}F$ / 0.9996
*CP*	$R_{-1}$	0.8717	$\mathcal{R}\mathcal{R}R_{-1}$ /0.9539	$\mathcal{R}\mathcal{R}R_{\frac{-1}{2}}$ / 0.9916
*CV*	$R_{\frac{-1}{2}}$	0.9095	$\mathcal{R}\mathcal{R}R_{\frac{-1}{2}}$ / 0.9967	$\mathcal{R}\mathcal{R}R_{\frac{-1}{2}}$ / 0.9968
*EM*	*ABC*	0.9145	$\mathcal{R}\mathcal{R}R_{\frac{-1}{2}}$ / 0.9979	$\mathcal{R}\mathcal{R}R_{\frac{-1}{2}}$ /0.9980
*HAC*	*ABC*	0.9167	$\mathcal{R}\mathcal{R}R_{\frac{-1}{2}}$ / 0.9979	$\mathcal{R}\mathcal{R}R_{\frac{-1}{2}}$ / 0.9980
*MR*	*ABC*	0.9176	$\mathcal{R}\mathcal{R}R_{\frac{-1}{2}}$ / 0.9978	$\mathcal{R}\mathcal{R}R_{\frac{-1}{2}}$ / 0.9979
*MW*	*ABC*	0.9146	$\mathcal{R}\mathcal{R}R_{\frac{-1}{2}}$ / 0.9979	$\mathcal{R}\mathcal{R}R_{\frac{-1}{2}}$ / 0.9980

In Table 6, we present the comparison of 16 degree based indices and 10 reduced reverse degree based indices against the properties of 26 benzenoid hydrocarbons along with their corresponding $R^{2}$ values. Based on the $R^{2}$ values corresponding to both the linear and quadratic regression models, it can be observed that the reduced reverse degree based indices are well correlated with the physico-chemical properties than the degree based indices. This implies that the reduced reverse degree-based descriptors are more suited for predicting the physico-chemical properties of benzenoid hydrocarbons compared to the existing degree based indices.

## Application to hyaluronic acid-paclitaxel conjugates

Cancer is one of the leading factors of mortality in the world, and its fatality rate is on the rise, with breast, stomach, lung, and colon cancers responsible for the bulk of deaths. Although there have been significant advancements in cancer biology and treatments. There are still challenges in the treatment of primary and metastatic disease. Furthermore, present anticancer medications cause limited selectivity and significant toxicity, greatly restricting their usefulness. In recent years, there have been some advancements in molecularly targeted anticancer therapy.

**Figure 7:**
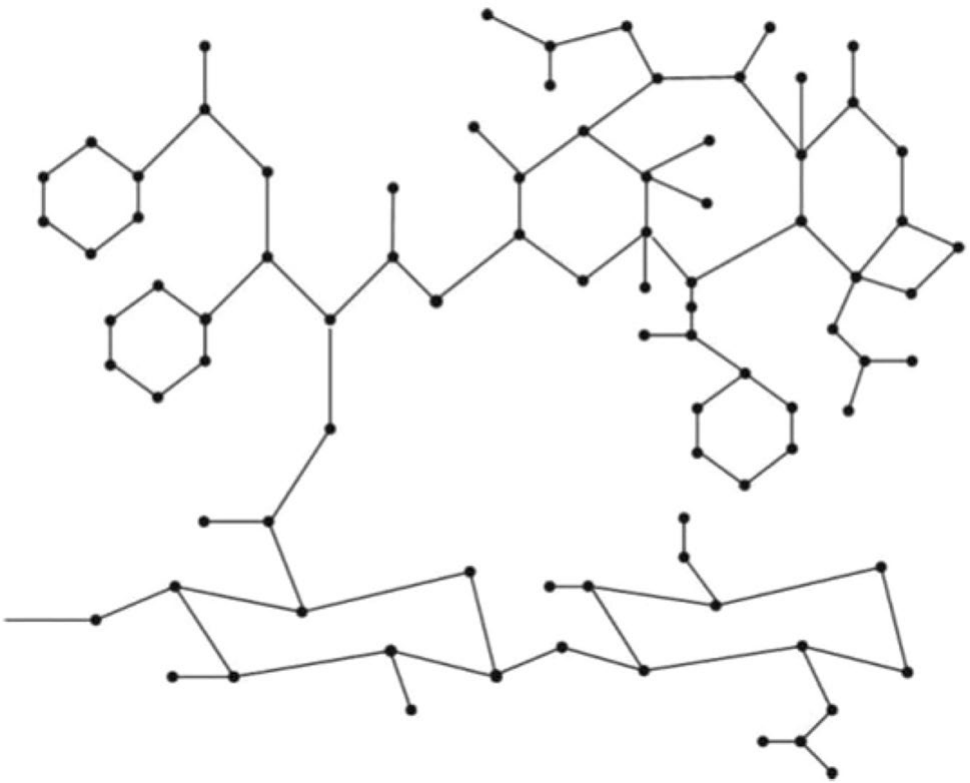
Molecular graph of $(HAP)_{n}$, $n=1$.

Hyaluronic acid (HA) is a compound that occurs naturally. It is a glycosaminoglycan polymer composed of a linear structure of units of D-glucuronic acid and N-acetyl-D-glucosamine. These are linked via alternating $\beta$-1,3- and $\beta$-1,4-glycosidic bonds. Disaccharide, HA’s primary structure is energetically stable^33^. Because of its unique biodegradable, biocompatible, harmless, hydrophilic, and non-immutable properties, HA is a prospective cancer treatment compound; also, HA receptors are over-expressed on numerous tumour cells. HA intends to improve antitumor therapeutics by targeting CD44-overexpressing cells, which is a rapidly developing platform nowadays^34–36^. HA is an excellent medication transporter and target.^37^. Paclitaxel (PTX) is an effective medication that is prescribed for a variety of malignancies, notably ovarian, breast, lung, bladder, prostate, and esophageal tumors. Although PTX treatment has its own set of drawbacks, such as poor solubility and associated adverse effects, as well as the excipients commonly utilised in its formulation. The key benefits of HA-PTX conjugates include increased water solubility and activity retention, as well as the possibility of using it as a drug carrier to boost anti-tumor potency^38–40^. Figure 7 shows the structure of hyaluronic acid-paclitaxel conjugates. Numerous degree-based indices of the Hyaluronic acid (HA) conjugates^41^ and Hyaluronic Acid-Paclitaxel conjugates^42–44^ have been proposed by researchers.

There are still no reduced reverse degree based topological results on the molecular structures of HA-Paclitaxel conjugates. As a result of the immense pharmaceutical significance of HA-Paclitaxel conjugates, present study intends to investigate the reduced reverse degree based topological indices of the chemical structure of HA-Paclitaxel conjugates. Furthermore, these findings may serve as a theoretical base for pharmacological engineering.

## Methodology and results

Figure 7 depicts the structure of the HA-Paclitaxel conjugates for the values $n = 1$. Edge partitioning based on the reduced reverse degree sum counting is the mechanism employed here. By observing the graph structure, we get $\mid V((HAP)_{n})\mid$ = 87*n* and $\mid E((HAP)_{n})\mid$ = 96*n*.

**Table 7:** Reduced reverse degree based edge partitions.

$E_{i}$	$(\mathcal{R}\mathcal{R}R(u), \mathcal{R}\mathcal{R}R(v))$	Number of edges
$E_{1}$	(5, 4)	$n+1$
$E_{2}$	(5, 3)	16*n*
$E_{3}$	(5, 2)	4*n*
$E_{4}$	(4, 4)	$13n+1$
$E_{5}$	(4, 3)	$32n-1$
$E_{6}$	(4, 2)	3*n*
$E_{7}$	(3, 3)	$19n-1$
$E_{8}$	(3, 2)	7*n*
$E_{9}$	(2, 2)	*n*

## Results on reduced reverse degree based descriptors of hyaluronic acid-paclitaxel conjugates

Here we compute the reduced reverse degree based topological indices of $(HAP)_{n}$, $n \ge 1$.

Applying the reduced reverse degree based edge partitions as given in Table 7, we get

$$\begin{aligned} \mathcal{R}\mathcal{R}M_{1}((HAP)_{n})&= \sum \limits _{uv \in E_{1}}\bigg [5+4\bigg ] + \sum \limits _{uv \in E_{2}}\bigg [5+3\bigg ] + \sum \limits _{uv \in E_{3}}\bigg [5+2\bigg ] + \sum \limits _{uv \in E_{4}}\bigg [4+4\bigg ] \\&\quad + \sum \limits _{uv \in E_{5}}\bigg [4+3\bigg ] + \sum \limits _{uv \in E_{6}}\bigg [4+2\bigg ] + \sum \limits _{uv \in E_{7}}\bigg [3+3\bigg ] + \sum \limits _{uv \in E_{8}}\bigg [3+2\bigg ] \\&\quad + \sum \limits _{uv \in E_{9}}\bigg [2+2\bigg ] \\&= 664n+4. \end{aligned}$$

$$\begin{aligned} \mathcal{R}\mathcal{R}M_{2}((HAP)_{n})&= \sum \limits _{uv \in E_{1}}\bigg [5*4\bigg ] + \sum \limits _{uv \in E_{2}}\bigg [5*3\bigg ] + \sum \limits _{uv \in E_{3}}\bigg [5*2\bigg ] + \sum \limits _{uv \in E_{4}}\bigg [4*4\bigg ] \\&\quad + \sum \limits _{uv \in E_{5}}\bigg [4*3\bigg ] + \sum \limits _{uv \in E_{6}}\bigg [4*2\bigg ] + \sum \limits _{uv \in E_{7}}\bigg [3*3\bigg ] + \sum \limits _{uv \in E_{8}}\bigg [3*2\bigg ] \\&\quad + \sum \limits _{uv \in E_{9}}\bigg [2*2\bigg ] \\&= 1133n+15. \end{aligned}$$

$$\begin{aligned} \mathcal{R}\mathcal{R}HM_{1}((HAP)_{n})&= \sum \limits _{uv \in E_{1}}\bigg [5+4\bigg ]^{2} + \sum \limits _{uv \in E_{2}}\bigg [5+3\bigg ]^{2} + \sum \limits _{uv \in E_{3}}\bigg [5+2\bigg ]^{2} \\&\quad + \sum \limits _{uv \in E_{4}}\bigg [4+4\bigg ]^{2} + \sum \limits _{uv \in E_{5}}\bigg [4+3\bigg ]^{2} + \sum \limits _{uv \in E_{6}}\bigg [4+2\bigg ]^{2} \\&\quad + \sum \limits _{uv \in E_{7}}\bigg [3+3\bigg ]^{2} + \sum \limits _{uv \in E_{8}}\bigg [3+2\bigg ]^{2} + \sum \limits _{uv \in E_{9}}\bigg [2+2\bigg ]^{2} \\&= 4684n+60. \end{aligned}$$

$$\begin{aligned} \mathcal{R}\mathcal{R}HM_{2}((HAP)_{n})&= \sum \limits _{uv \in E_{1}}\bigg [5*4\bigg ]^{2} + \sum \limits _{uv \in E_{2}}\bigg [5*3\bigg ]^{2} + \sum \limits _{uv \in E_{3}}\bigg [5*2\bigg ]^{2} + \sum \limits _{uv \in E_{4}}\bigg [4*4\bigg ]^{2} \\&\quad + \sum \limits _{uv \in E_{5}}\bigg [4*3\bigg ]^{2} + \sum \limits _{uv \in E_{6}}\bigg [4*2\bigg ]^{2} + \sum \limits _{uv \in E_{7}}\bigg [3*3\bigg ]^{2} + \sum \limits _{uv \in E_{8}}\bigg [3*2\bigg ]^{2} \\&\quad + \sum \limits _{uv \in E_{9}}\bigg [2*2\bigg ]^{2} \\&= 14335n+431. \end{aligned}$$

$$\begin{aligned} \mathcal{R}\mathcal{R}F((HAP)_{n})&= \sum \limits _{uv \in E_{1}}\bigg [5^{2}+4^{2}\bigg ] + \sum \limits _{uv \in E_{2}}\bigg [5^{2}+3^{2}\bigg ] + \sum \limits _{uv \in E_{3}}\bigg [5^{2}+2^{2}\bigg ] \\&\quad + \sum \limits _{uv \in E_{4}}\bigg [4^{2}+4^{2}\bigg ] + \sum \limits _{uv \in E_{5}}\bigg [4^{2}+3^{2}\bigg ] + \sum \limits _{uv \in E_{6}}\bigg [4^{2}+2^{2}\bigg ] \\&\quad + \sum \limits _{uv \in E_{7}}\bigg [3^{2}+3^{2}\bigg ] + \sum \limits _{uv \in E_{8}}\bigg [3^{2}+2^{2}\bigg ] + \sum \limits _{uv \in E_{9}}\bigg [2^{2}+2^{2}\bigg ] \\&= 2418n+30. \end{aligned}$$

$$\begin{aligned} \mathcal{R}\mathcal{R}ABC((HAP)_{n})&= \sum \limits _{uv \in E_{1}}\bigg [\sqrt{\frac{5+4-2}{5*4}}\bigg ] + \sum \limits _{uv \in E_{2}}\bigg [\sqrt{\frac{5+3-2}{5*3}}\bigg ] \\&\quad + \sum \limits _{uv \in E_{3}}\bigg [\sqrt{\frac{5+2-2}{5*2}}\bigg ] + \sum \limits _{uv \in E_{4}}\bigg [\sqrt{\frac{4+4-2}{4*4}}\bigg ] \\&\quad + \sum \limits _{uv \in E_{5}}\bigg [\sqrt{\frac{4+3-2}{4*3}}\bigg ] + \sum \limits _{uv \in E_{6}}\bigg [\sqrt{\frac{4+2-2}{4*2}}\bigg ] \\&\quad + \sum \limits _{uv \in E_{7}}\bigg [\sqrt{\frac{3+3-2}{3*3}}\bigg ] + \sum \limits _{uv \in E_{8}}\bigg [\sqrt{\frac{3+2-2}{3*2}}\bigg ] + \sum \limits _{uv \in E_{9}}\bigg [\sqrt{\frac{2+2-2}{2*2}}\bigg ] \\&= 62.6009177191n-0.1082. \end{aligned}$$

$$\begin{aligned} \mathcal{R}\mathcal{R}GA((HAP)_{n})&= \sum \limits _{uv \in E_{1}}\bigg [\frac{2*\sqrt{5*4}}{5+4}\bigg ] + \sum \limits _{uv \in E_{2}}\bigg [\frac{2*\sqrt{5*3}}{5+3}\bigg ] + \sum \limits _{uv \in E_{3}}\bigg [\frac{2*\sqrt{5*2}}{5+2}\bigg ] \\&\quad + \sum \limits _{uv \in E_{4}}\bigg [\frac{2*\sqrt{4*4}}{4+4}\bigg ] + \sum \limits _{uv \in E_{5}}\bigg [\frac{2*\sqrt{4*3}}{4+3}\bigg ] + \sum \limits _{uv \in E_{6}}\bigg [\frac{2*\sqrt{4*2}}{4+2}\bigg ] \\&\quad + \sum \limits _{uv \in E_{7}}\bigg [\frac{2*\sqrt{3*3}}{3+3}\bigg ] + \sum \limits _{uv \in E_{8}}\bigg [\frac{2*\sqrt{3*2}}{3+2}\bigg ] + \sum \limits _{uv \in E_{9}}\bigg [\frac{2*\sqrt{2*2}}{2+2}\bigg ] \\&= 93.9919881117n+1.9836. \end{aligned}$$

$$\begin{aligned} \mathcal{R}\mathcal{R}R_{\alpha }((HAP)_{n})&= \sum \limits _{uv \in E_{1}}\bigg [5*4\bigg ]^{\alpha } + \sum \limits _{uv \in E_{2}}\bigg [5*3\bigg ]^{\alpha } + \sum \limits _{uv \in E_{3}}\bigg [5*2\bigg ]^{\alpha } + \sum \limits _{uv \in E_{4}}\bigg [4*4\bigg ]^{\alpha } \\&\quad + \sum \limits _{uv \in E_{5}}\bigg [4*3\bigg ]^{\alpha } + \sum \limits _{uv \in E_{6}}\bigg [4*2\bigg ]^{\alpha } + \sum \limits _{uv \in E_{7}}\bigg [3*3\bigg ]^{\alpha } + \sum \limits _{uv \in E_{8}}\bigg [3*2\bigg ]^{\alpha } \\&\quad + \sum \limits _{uv \in E_{9}}\bigg [2*2\bigg ]^{\alpha }. \end{aligned}$$

In the above $\mathcal{R}\mathcal{R}R_{\alpha }((HAP)_{n})$,

- If $$\alpha = \frac{1}{2}$$, then $$\mathcal{R}\mathcal{R}R_{\alpha }((HAP)_{n})$$ = 326.571942n+2.00803434.
- If $$\alpha = -1$$, then $$\mathcal{R}\mathcal{R}R_{\alpha }((HAP)_{n})$$ = 8.8986n-0.0819.
- If $$\alpha = \frac{-1}{2}$$, then $$\mathcal{R}\mathcal{R}R_{\alpha }((HAP)_{n})$$ = 26.1823n-0.0075.

## Conclusion

In this article, novel topological descriptors $\mathcal{R}\mathcal{R}M_{1}$, $\mathcal{R}\mathcal{R}M_{2}$, $\mathcal{R}\mathcal{R}HM_{1}$, $\mathcal{R}\mathcal{R}HM_{2}$, $\mathcal{R}\mathcal{R}F$, $\mathcal{R}\mathcal{R}ABC$, $\mathcal{R}\mathcal{R}GA$ and $\mathcal{R}\mathcal{R}R_{\alpha }$ have been analyzed with respect to 26 Benzenoid Hydrocarbons. From the QSPR analysis, it is evident that these descriptors are useful molecular descriptors. We tested the predictive capability of the indices with respect to 26 Benzenoid Hydrocarbons.

QSPR study using curvilinear models reveals that both linear and quadratic regression models provide good estimates for the physico-chemical properties of the 26 Benzenoid Hydrocarbons. From quadratic regression models, we observe that our proposed indices have high correlation with all the physico-chemical properties considered in the above sections.

The best models that predict the physico-chemical properties are as follows:

1. $\mathcal{R}\mathcal{R}R_{-1}$ is best suited for predicting the properties, *BP*, *LogP*.
2. $\mathcal{R}\mathcal{R}R_{\frac{-1}{2}}$ is best suited for predicting the properties, *CP*, *CV*, *EM*, *HAC*, *MR* and *MW*.
3. $RRHM_{1}$ is best suited for predicting *GE*.
4. *RRGA* is best suited for predicting *CT*.
5. *RRABC* is best suited for predicting *PI*.
6. *RRF* is best suited for predicting *HL*.

On comparing the proposed reduced reverse degree-based indices with the existing degree-based topological indices, we found that our proposed indices are well correlated with all the physico-chemical properties of 26 benzenoid hydrocarbons. The errors are significantly reduced for our proposed indices. This demonstrates the significance of the reduced reverse degree-based descriptors in predicting the physico-chemical properties of benzenoid hydrocarbons over the existing degree-based descriptors.

The defined reduced reverse degree based topological indices have been determined for Hyaluronic Acid-Paclitaxel Conjugates $(HAP)_{n}$, $n \ge 1$.

In future, these indices can be applied to various transformations of graphs and to analyze different chemical networks.

## Data Availability

All data generated or analysed during this study are included in this published article. The experimental data for benzenoid hydrocarbons were taken from https://pubchem.ncbi.nlm.nih.gov. MS-Excel 2019 was used for statistical analyses.
